# Datasets from harmonised metabolic phenotyping of root, tuber and banana crop

**DOI:** 10.1016/j.dib.2022.108041

**Published:** 2022-03-12

**Authors:** Margit Drapal, Laura Perez-Fons, Elliott J. Price, Delphine Amah, Ranjana Bhattacharjee, Bettina Heider, Mathieu Rouard, Rony Swennen, Luis Augusto Becerra Lopez-Lavalle, Paul D. Fraser

**Affiliations:** 1 Royal Holloway University of London, Surrey, TW20 0EX, United Kingdom; 2 International Institute of Tropical Agriculture, PMB 5320, Ibadan, Nigeria; 3 International Potato Center, La Molina, CP 1558, Lima, Peru.; 4 Bioversity International, Parc Scientifique Agropolis II, 34397 Montpellier, France; 5 Laboratory of Tropical Crop Improvement, Division of Crop Biotechnics, KU Leuven, B-3001 Leuven, Belgium; 6 Bioversity International, Willem De Croylaan 42, B-3001 Leuven, Belgium; 7 International Institute of Tropical Agriculture. C/0 The Nelson Mandela African Institution of Science and Technology, P.O. Box 44, Arusha, Tanzania; 8 International Center for Tropical Agriculture, Cali, CP 763537, Colombia

**Keywords:** Metabolomics, underutilised crops, banana, cassava, sweet potato, yam, potato

## Abstract

Biochemical characterisation of germplasm collections and crop wild relatives (CWRs) facilitates the assessment of biological potential and the selection of breeding lines for crop improvement. Data from the biochemical characterisation of staple root, tuber and banana (RTB) crops, i.e. banana (*Musa* spp.), cassava (*Manihot esculenta*), potato (*Solanum tuberosum*), sweet potato (*Ipomoea batatas*) and yam (*Dioscorea spp*.), using a metabolomics approach is presented. The data support the previously published research article “Metabolite database for root, tuber, and banana crops to facilitate modern breeding in understudied crops” (Price et al., 2020) [1].

Diversity panels for each crop, which included a variety of species, accessions, landraces and CWRs, were characterised. The biochemical profile for potato was based on five elite lines under abiotic stress. Metabolites were extracted from the tissue of foliage and storage organs (tuber, root and banana pulp) via solvent partition. Extracts were analysed via a combination of liquid chromatography – mass spectrometry (LC-MS), gas chromatography (GC)-MS, high pressure liquid chromatography with photodiode array detector (HPLC-PDA) and ultra performance liquid chromatography (UPLC)-PDA. Metabolites were identified by mass spectral matching to in-house libraries comprised from authentic standards and comparison to databases or previously published literature.

## Specifications Table

**Table:** 

Subject	Omics: Metabolomics
Specific subject area	Metabolite profiling of roots, tuber and bananas
Type of data	XLSX format
How the data were acquired	Mass spectrometry data were obtained with two analytical platforms: - Dionex Ultimate 3000 UHPLC (Thermo Scientific) coupled to maxis Ultra-High Resolution QTOF (Bruker, Germany) with ESI in negative ionisation mode - 7890A GC on-line with 5975C MSD (Agilent Technologies, US) - Analysis for carotenoids and chlorophylls was performed with Acquity UPLC-PDA (Waters, UK) or Alliance HPLC-PDA (Waters, UK)
Data format	Raw (LC-MS data) Analysed (tabulated format of metabolites identified by GC-MS) Processed/Filtered (database of identified metabolites)
Description of data collection	Lyophilised and ground plant tissue was extracted with a methanol/water or methanol/100mM Tris-HCl with 1M NaCl and chloroform method (1:1:2, vol.). Carotenoid/chlorophylls were analysed by HPLC-PDA or UPLHC-PDA analysis. Metabolite profiling of the aqueous and organic phase was performed with GC-MS and LC-MS. GC-MS raw data was processed with AMDIS and LC-MS data files were analysed with metaMS package in R. Data was normalised relative to the internal standard and to the sample weight (μg/g dry weight) and batch correction with QC was applied with large sample sets.
Data source location	Royal Holloway University of London, Egham, United Kingdom
Data accessibility	Database is published in Price *et al.* (2020). Pre-processed data is available at Mendeley Data for sweet potato [2], potato [3], banana [4] and cassava [5], [6], [7]. GC-MS data files are available for yam [8].
Related research article	E.J. Price, M. Drapal, L. Perez-Fons, D. Amah, R. Bhattacharjee, B. Heider, M. Rouard, R. Swennen, L.A. Becerra Lopez-Lavalle, P.D. Fraser, Metabolite database for root, tuber, and banana crops to facilitate modern breeding in understudied crops, Plant J. 101 (2020) 1258–1268. https://doi.org/10.1111/tpj.14649.

## Value of the Data

• The database provides a valuable resource describing the biochemical composition of cassava, sweet potato, potato, yam and banana.
• The database can be used to compare chemotypes of varieties/species of root, tuber and banana crops.
• The database can facilitate the identification of agronomic and consumer traits with quantifiable biochemical markers.
• Specific biochemical signatures can be identified for breeding selection.

## 1 Data Description

Resources for genetic and phenotypic diversity in underutilised crops are an important aspect for successful breeding efforts. Analysis of the metabolite composition of respective tissues/crops enables the assessment of chemical diversity available, the identification of certain phenotypes (e.g. nutrients content) or the elucidation of underlying mechanisms for specific traits (e.g. whitefly resistance in cassava [9]). As part of the Roots, Tubers and Bananas (RTB) project, metabolomics was used to analyse diversity panels of 38 banana accessions [10], 23 cassava varieties [11], 25 sweet potato accessions [12] and five yam species (*D. rotundata, D. cayenensis, D. alata, D. bulbifera* and *D. dumetorum*) [13], [14], [15]. In addition, five potato varieties were analysed to identify metabolites associated with resistance to drought [16] and two cassava varieties were compared to characterise the natural variation in resistance to whitefly [9]. Analysis of these crops was performed on different plant tissues (e.g. leaf, tuber and root) and for banana and cassava, on plants under two different cultivation conditions: *in vitro* propagation and open field.

**Fig. 1:**
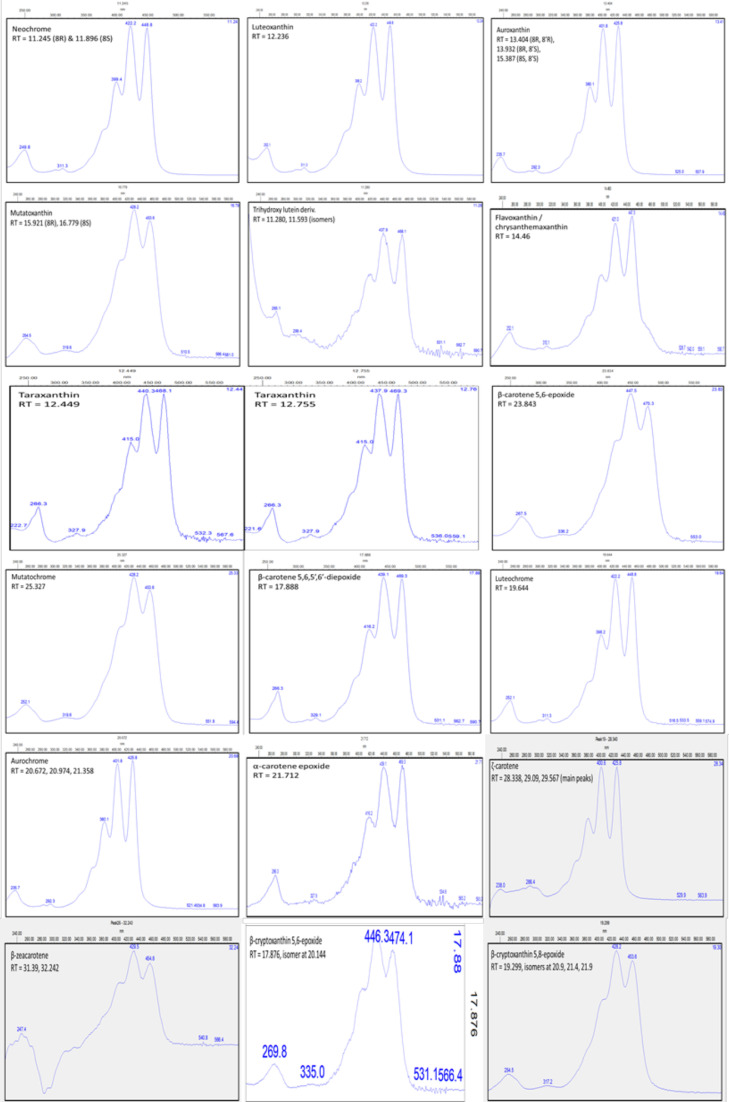
UV/Vis spectra of carotenoids and xanthophylls. The respective names of the compounds is displayed at the bottom right side of the spectrum. Retention times (RT) are listed as minutes underneath the compound name. Numbers in the spectra indicate the wavelength of the peaks characteristic for the respective compounds.

The respective species/accessions were subjected to a standard methanol-water-chloroform extraction, followed by different metabolomics techniques. LC-MS and GC-MS analysis was performed for untargeted profiling of polar and non-polar extracts. Analysis of non-polar extracts by LC-PDA was performed for a more targeted screening of isoprenoids (e.g. carotenoids and chlorophylls). Compounds in the samples were compared to retention time and UV/Vis spectrum of authentic standards (Fig. 1).

Molecular features detected in the different analysis techniques, were compared to authentic standards and spectral features in databases (e.g. NIST) for metabolite identification. For GC-MS, Automated mass spectral deconvolution and identification system (AMDIS) was used and settings were modified specific to certain crops (Table 1). For LC-MS analysis, the R package metaMS was used and a script for molecular feature extraction and library comparison was modified for samples analysed with maXis Ultra-High Resolution QTOF (Bruker, Germany). The outputs from both analysis techniques are available, as unprocessed Excel tables listing the areas of individual molecular features/metabolites in the respective samples, in Mendeley Data repository [2], [3], [4], [5], [6], [7], [8]. The identified metabolites were quantified relative to internal standards. A database was compiled for the present RTB crops and includes the quantitative range of each metabolite present in the individual tissues of each genus [1].

**Table 1:** Settings for Automated mass spectral deconvolution and identification system (AMDIS) for data analysis of GC-MS files.

AMIDS Settings	Yam (polar)	Yam (non-polar)	Cassava	Potato, sweet potato and banana
**Identification**				
Minimum match factor	—————————————- 80 —————————————–			
Multiple identification per compound	—————————————- yes —————————————-			
Show standards	—————————————- no —————————————–			
Only reverse search	—————————————- no —————————————–			
Type of analysis	————————– Use retention index data ————————–			
RI window	9+0*0.01	9+0*0.01	1+0*0.01	20+0*0.01
Match factor penalties level	average	average	strong	average
Max. penalty	20	20	10	20
No RI in library	10	10	10	10

**Instrument**				
Low m/z	———————————— auto (50) ————————————			
High m/z	———————————— auto (520) ———————————–			
Use scan set	—————————————- no —————————————–			
Threshold	————————————— high —————————————			
Scan direction	———————————— high to low ———————————-			
Data file format	———————————– Agilent files ———————————-			
Instrument type	———————————– Quadrupole ———————————-			

**Deconvolution**				
Component width	12	12	32	32
Omitted	—————————————- 28 —————————————–			
Adjacent peak subtraction	two	two	one	two
Resolution	low	low	low	medium
Sensitivity	very low	low	very low	low
Shape	high	medium	medium	low

**QA/QC**				
Solvent tailing (m/z)	—————————————- 84 —————————————–			
Column bleed (m/z)	—————————————- 207 —————————————–			

## 2 Experimental Design, Materials and Methods

### 2.1 Metabolite extraction

Lyophilised tissue was ground and homogenised to a fine powder. Aliquots (10 mg) were weighed for each sample and extracted with a methanol/water/chloroform extraction method. Due to the size of the individual sample sets, sample batch of 22 sample were created. Each sample batch included an extraction blank and a quality control, which represented a pool of a samples in the respective sample set. Extraction methods were optimised for specific chemical classes and for each crop [10], [11], [12], [13], [14], [15], [16], [17], [18], e.g. carotenoid extraction for yam with 200 mg/sample. The yam dataset was created with GC-MS analysis of aqueous and organic phase and HPLC-DAD analysis of the organic phase. Datasets for all other crops (sweet potato, potato, banana and cassava) were created with GC-MS and LC-MS analysis of the aqueous phase and UPLC-DAD analysis of the organic phase. The dataset for sweet potato and cassava also included GC-MS analysis of the organic phase.

### 2.2 Liquid chromatography-mass spectrometry (LC-MS) analysis

**Fig. 2:**
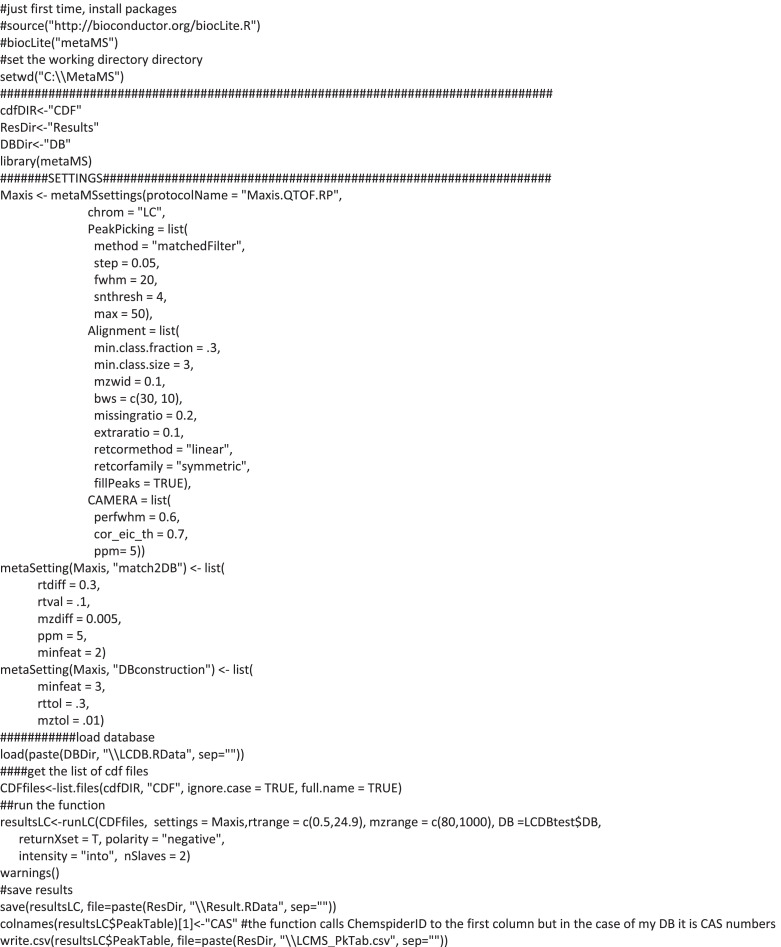
R script for R package metaMS to convert raw LC-MS files into an unprocessed Excel file.

Aqueous extracts were dried down and resuspended in methanol/water (1:1, 100 μL). Internal standard (homogentisic acid, 5 μg, or genistein, 10 μg) was added to each sample, the extraction blank and the quality control. Samples were filtered (nylon, 0.45 μm) and analysed with Dionex Ultimate 3000 UHPLC (Thermo Scientific) coupled to maXis Ultra-High Resolution QTOF (Bruker, Germany) in negative electrospray ionisation mode (Vi, 5 μl). Aliquots of samples (10 μL) were separated with Acquity BEH C18 column and a solvent gradient including 0.1% formic acid in water and acetonitrile [11]. Extraction of chemical features from raw data files and search chemical database was compiled with R package metaMS [19,20] (Fig. 2) including an in-house library with authentic standards. Identification was set to m/z difference 0.005 and retention time difference 0.3 min. The resulting data matrix containing integrated peak areas of both unidentified chemical features and annotated metabolites was exported as Microsoft Excel Open XML Spreadsheet (.xlsx) format.

### 2.3 Gas chromatography-mass spectrometry (GC-MS) analysis

Dried extracts were derivatised with methoxyamine hydrochloride (MeOX) in pyridine, followed by *N-*methyl-*N*-(trimethylsilyl)trifluoroacetamine (MSTFA) at 40°C. The 7890A GC on-line with 5975C MSD (Agilent Technologies, US) was set with 1 μL injection volume in splitless mode and a temperature gradient from 70-325°C [13] using a DB-5MS column.

Data was compiled with AMDIS (v2.71, NIST) and an in-house library specific to each crop. Deconvolution and identification settings were optimised for each crop (Table 1).

### 2.4 High pressure and ultra performance liquid chromatography (HPLC/UPLC) analysis

Both HPLC and UPLC analysis included a photodiode array detector (PDA). Extracts were dried down and resuspended in ethyl acetate, to concentrate the amount of carotenoids and chlorophylls present. For analysis of yam samples by Alliance HPLC-PDA (Waters, UK), an aliquot (20μL) was injected and separated on a reverse-phase (RP) column (4.6 × 250 mm, C_30_, 5μm particle size; YMC Inc., Kyoto, Japan) at 25°C with a 60min solvent gradient including three buffers [14]. For all other crops, Acquity UPLC-PDA was employed with lower injection volume (3-7μL), an Ethylene Bridged Hybrid (BEH C_18_) column (2.1 × 100mm, 1.7μm) with a BEH C_18_ VanGuard precolumn (2.1 × 50mm, 1.7μm) and a mobile phase consisting of two buffers [21]. The PDAs were scanning in a continuous manner from 250-600nm.

Peaks were integrated using Empower 2 (Waters, UK) and identified through chromatographic and spectral characteristics to standards (Fig. 1) and literature references [22].

### 2.5 Data processing

Data output from the respective data analysis software was tabulated using IdAlign (Centre for Computational Systems Biology, University of Western Australia, http://www.softsea.com/review/IdAlign.html) and Microsoft Excel 2016. Metabolite features present in extraction blanks were subtracted or excluded from the data sets. The identified metabolites and molecular features were quantified relative to the respective internal standard and the datasets were normalised to the individual sample weights (μg/g dry weight). Some compounds were detected as multiple derivatives (e.g. glutamic acid and pyroglutamic acid) and their areas were merged before normalisation. In some cases, the data needed to be normalised with the quality controls to correct for batch effects (e.g. cassava [11]).

## Ethics statements

This work included plant material and did not include work involved with human subjects, animal experiments or data collected from social media platforms.

## CRediT Author Statement

**Margit Drapal, Laura Perez** and **Elliott Price:** Data generation and curation, Writing – original draft preparation; **Delphine Amah, Ranjana Bhattacharjee, Bettina Heider, Mathieu Rouard, Rony Swennen** and **Luis Augusto Becerra Lopez-Lavalle:** Provided germplasm; **Paul D. Fraser:** Conceptualization, Supervision and Writing – review & editing.

## Declaration of Competing Interest

The authors declare that they have no known competing financial interests or personal relationships that could have appeared to influence the work reported in this paper.
